# Prevalence and genetic variants of G6PD deficiency among two Malagasy populations living in *Plasmodium vivax*-endemic areas

**DOI:** 10.1186/s12936-017-1771-6

**Published:** 2017-04-04

**Authors:** Rosalind E. Howes, Ernest R. Chan, Tovonahary Angelo Rakotomanga, Seth Schulte, John Gibson, Melinda Zikursh, Thierry Franchard, Brune Ramiranirina, Arsène Ratsimbasoa, Peter A. Zimmerman

**Affiliations:** 1 Center for Global Health and Diseases, Case Western Reserve University, Cleveland, OH USA; 2 Nuffield Department of Medicine, Oxford Big Data Institute, University of Oxford, Oxford, UK; 3 Institute for Computational Biology, Case Western Reserve University, Cleveland, OH USA; 4 National Malaria Control Programme, Ministry of Health, Antananarivo, Madagascar; 5 Faculty of Science, University of Antananarivo, Antananarivo, Madagascar; 6 University of Antananarivo, Antananarivo, Madagascar

**Keywords:** Madagascar, Glucose-6-phosphate dehydrogenase deficiency, G6PDd genotypes, Primaquine, *Plasmodium vivax*

## Abstract

**Background:**

The prevalence and variants of G6PD deficiency in the *Plasmodium vivax*-endemic zones of Madagascar remain unknown. The admixed African-Austronesian origins of the Malagasy population make it probable that a heterogeneous mix of genetic variants with a spectrum of clinical severity will be circulating. This would have implications for the widespread use of *P. vivax* radical cure therapy. Two study populations in the *P. vivax*-endemic western foothills region of Madagascar were selected for G6PD screening. Both the qualitative fluorescent spot test and G6PD genotyping were used to screen all participants.

**Results:**

A total of 365 unrelated male volunteers from the Tsiroanomandidy, Mandoto, and Miandrivazo districts of Madagascar were screened and 12.9% were found to be phenotypically G6PD deficient. Full gene sequencing of 95 samples identified 16 single nucleotide polymorphisms, which were integrated into a genotyping assay. Genotyping (n = 291) found one individual diagnosed with the severe *G6PD Mediterranean* ^*C563T*^ mutation, while the remaining G6PD deficient samples had mutations of African origin, *G6PD A*- and *G6PD A*.

**Conclusions:**

Deployment of *P. vivax* radical cure in Madagascar must be considerate of the risks presented by the observed prevalence of G6PDd prevalence. The potential morbidity associated with cumulative episodes of *P. vivax* clinical relapses requires a strategy for increasing access to safe radical cure. The observed dominance of African G6PDd haplotypes is surprising given the known mixed African-Austronesian origins of the Malagasy population; more widespread surveying of G6PDd epidemiology across the island would be required to characterize the distribution of G6PD haplotypes across Madagascar.

## Background

Malaria remains a major public health burden in Madagascar, with the entire Malagasy population living at risk of infection and suffering an estimated 2.4 million (1.5–4.0 million) cases of malaria in 2015 [1]. Temporal trends in reported health facility data suggest significant increases in malaria burden from 2010 to 2015 [2]. The last major national review of the health status of the Malagasy population reported malaria to be the second most common cause of death in under-5 year olds in district hospitals, and the fourth leading cause of all outpatient consultations across all age groups [3]. The island is biogeographically diverse, with varied malaria transmission patterns across different parts of the island ranging from epidemic, to highly seasonal, to sustained year-round transmission [2]. Malaria in Madagascar is predominantly caused by *Plasmodium falciparum*, though the other human malaria species are also present [4, 5] and likely under-reported. *Plasmodium vivax* transmission is reported from sites across the island, but most commonly in the fringe zone between the west coast and the central highlands [6, 7]. Parasites in these regions were found to be infecting and causing clinical *P. vivax* malaria in Duffy negative individuals, the blood group previously considered refractory to infection due to the absence of Duffy antigen expression on the surface of red blood cells [6, 8, 9]. A cross-sectional study using PCR-based molecular diagnosis in this western highland fringe region found an 8% prevalence of *P. vivax*, the same as that for *P. falciparum* [10].

Madagascar’s current national strategic plan (2013–2017) calls for dual applications of the anti-malarial primaquine: (i) in low dose regimens for blocking *P. falciparum* transmission in epidemic-prone zones and in low transmission areas targeting pre-elimination status, and (ii) in its 14-day formulation as *P. vivax* radical cure [11]. However, neither application of primaquine is deployed, in large part due to uncertainties relating to safety in glucose-6-phosphate dehydrogenase deficient (G6PDd) patients (National Malaria Control Programme, Pers. Comm.) [12].

G6PD deficiency is highly diverse, with at least 217 genetic variants expressing a spectrum of enzyme phenotypes of differing susceptibilities to toxic substances including primaquine [13]. At the global scale, distinct regional patterns of G6PDd epidemiology emerge [14]. Given the contemporary Malagasy population’s predominantly African and Austronesian origins [15], it could be anticipated that a unique combination of alleles originating from these regions may be present. G6PD deficiency tends to be more common but less clinically severe and heterogeneous in African populations than in Asian [14]. For instance, 27 African countries are predicted to have mean frequencies of >10% in males, while prevalence is estimated at 7.1% in Indonesian males (50% CI 5.3–9.4%) [16]. A single *G6PD*d allelic variant predominates in African communities (*G6PD A*-^*202A/376G*^) which is generally less severe than most of the diverse variants found in Asian populations (including, among many, *G6PD Viangchan* ^*871A*^, *G6PD Vanua Lava* ^*383C*^ and *G6PD Chatham* ^*1003A*^) [17, 18].

Recognizing the uncertainty around the potentially complex *G6PD* allele inheritance in Madagascar, this study therefore aims to begin strengthening the evidence base of epidemiological data available to the Malagasy National Malaria Control Programme (NMCP) regarding G6PD deficiency. Providing this insight will contribute to improved case management by increasing access to safe primaquine treatment, notably in its higher dosing for *P. vivax* radical cure. This study directly addresses a stated operational research priority in the country’s National Strategic Plan (2013–2017). To date, the only published survey of the Malagasy population was from the east coast city of Manakara in 1966 which found 21% of males with G6PDd (n tested = 70) [19].

## Methods

### Ethics statement

This study protocol was approved by the ethical review panels of University Hospitals Case Medical Center, Cleveland, Ohio, USA and the National Institutes of Health, USA (DMID Protocol #13-0067), and the Ministry of Public Health, Madagascar (Nº099-MSANP/CE). Written informed consent was obtained from all subjects, or subject guardians, enrolled into the study.

### Study populations

**Fig. 1:**
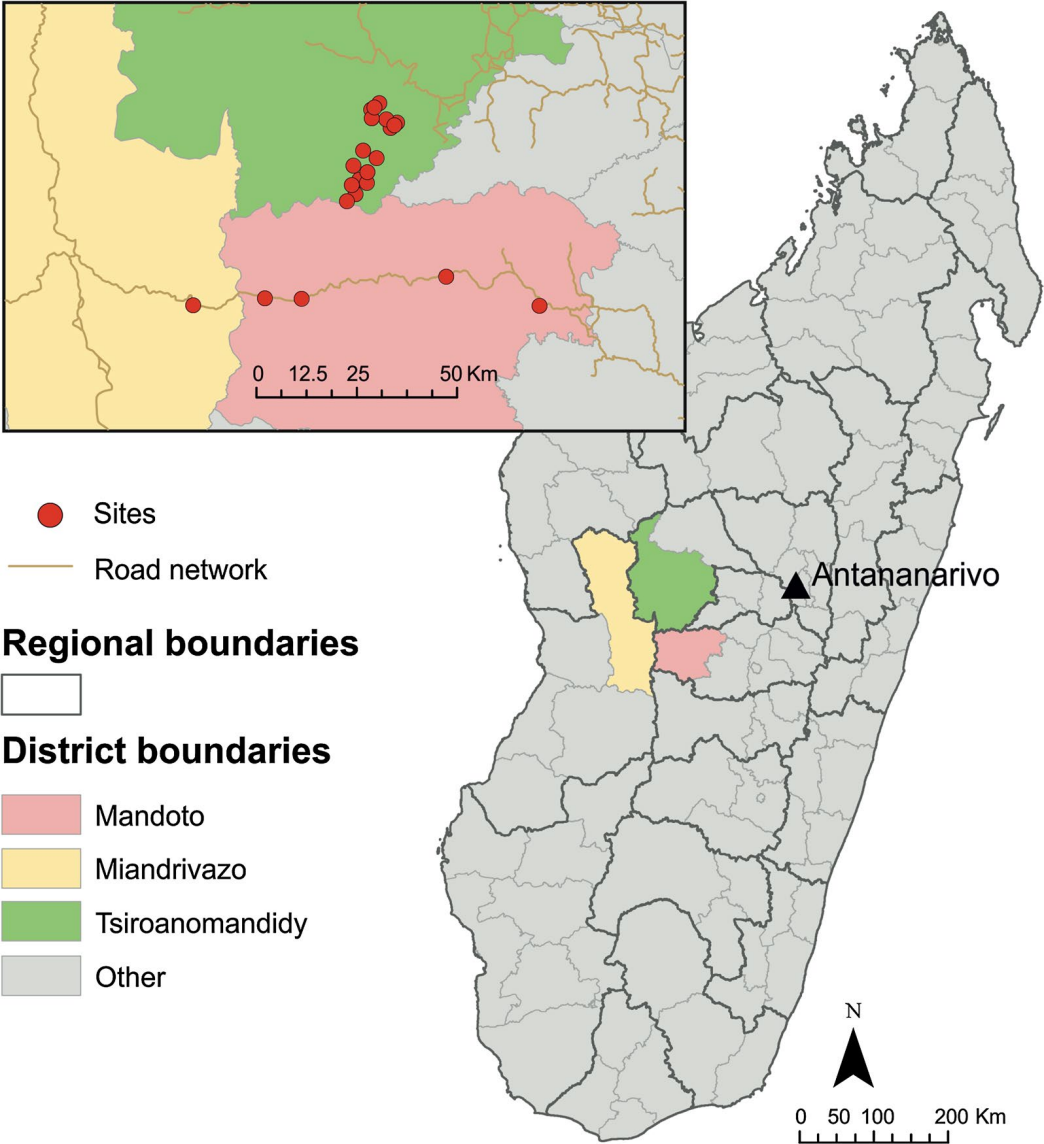
Study region map. *Red points* mark the villages surveyed. Antananarivo (*black triangle*) is the capital of Madagascar, from where the community surveyed in the Ampasimpotsy community of Tsiroanomandidy district were originally from

Two populations were surveyed which were considered genetically representative of populations targeted for the two regimens of primaquine therapy (Fig. 1). First, rural communities resident in the *P. vivax*-endemic highland fringe ecozone of Mandoto and Miandrivazo districts were surveyed. Second, recruitment was conducted among a migrant population resident in the rural Ampasimpotsy community of Tsiroanomandidy district where *P. vivax* is also endemic [6]. This migrant community was recently (within at most one generation) relocated from urban Antananarivo in the central highlands. The central highlands are an area where single-dose primaquine interventions are scheduled to prevent *P. falciparum* transmission and reinforce elimination efforts [11]. This recently relocated population was therefore considered equivalent to a population sample from the city of Antananarivo itself.

### Sampling method

To facilitate diagnosis of G6PD enzyme activity and increase the reliability of this study’s prevalence estimates, only males were surveyed. The *G6PD* gene’s position on the X-chromosome means that allele frequency corresponds directly to the prevalence of deficiency in males, though not in females [14, 20]. Sampling was random and included males of all age groups. To limit double sampling of genetically related individuals, a single individual per family was included. A census was available for ensuring that individuals surveyed were consistent with this collection strategy in the Ampasimpotsy region, while in Mandoto/Miandrivazo districts this relied on the enrolment interviews. Demographic information was obtained for all participants by questionnaire. Sampling took place in Mandoto and Miandrivazo districts in July 2015, and in Ampasimpotsy in June and August 2015.

**Fig. 2:**
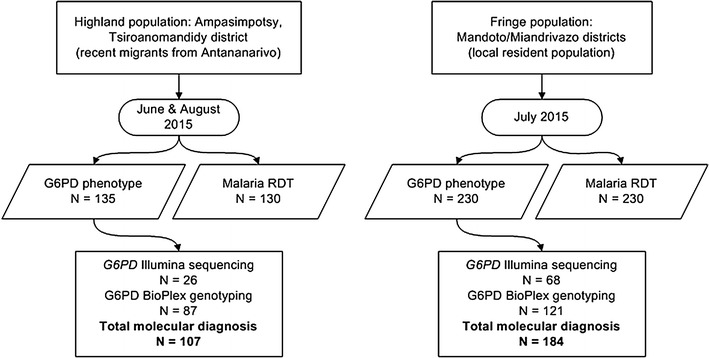
Study sampling design

*G6PD*d allele frequency in Madagascar has been predicted to be around 19.4% (50% CI 11.5–30.3%) [16]. A sample size of 235 was determined from Mandoto/Miandrivazo districts to ensure 95% confidence around a 5% precision interval of the G6PDd prevalence estimate [21]. The smaller population size and high degree of relatedness within the closed migrant community in Ampasimpotsy constrained the sample size to 135, which corresponded to approximately 90% confidence level in the estimate. The study design is summarised in Fig. 2.

### Field-based blood processing

All assays used finger-prick capillary blood [22]. First, rapid diagnosis of *Plasmodium* blood-stage parasitaemia performed using the CareStartTM Malaria HRP2/pLDH(Pf/PAN) Combo assay (AccessBio, Seoul, Korea), according to the manufacturer’s instructions. Second, the fluorescent spot test (catalogue number 203-A; Trinity Biotech, Wicklow, Ireland) was used for qualitative assessment of G6PD enzyme activity. Finally, 200 µL of blood was collected into K^+^EDTA microtainers for molecular diagnosis.

### Qualitative G6PD phenotype field-based assay

The fluorescent spot test (FST) was used according to the manufacturer’s recommended protocol with minor modifications as described here. Prior to field studies, diagnostic performance was compared between the manufacturer’s protocol and the conditions anticipated during field trials using the normal, intermediate, and deficient controls provided by the supplier (catalogue numbers G6888, G5029, and G5888, respectively). First, to address the lack of electricity to power incubators in the field, controlled laboratory tests varied the incubation temperature (37 °C vs ambient temperature range in the field [19–31 °C]) and assessed the impact on the test reaction outcomes. Incubation of samples from the supplier’s controls at ambient field site temperatures instead of 37 °C resulted in all samples (n = 9) being correctly diagnosed (“normal”, “intermediate” or “deficient”) by visual qualitative assessment under UV light following incubation at the lower temperatures. Furthermore, in the field, controls were run at several time points during the day to reflect the fluctuating ambient temperatures, and diagnoses were based on comparison with the fluorescence intensities of controls performed during the same time/temperature window. Second, diagnostic test volumes of blood and reactant were experimentally reduced to 50 and 25% of the protocol-recommended volumes to allow a greater number of tests to be performed. When reaction volumes were reduced to 25% of recommended volumes, it was not possible to distinguish G6PD normal from deficient blood as no fluorescence could be seen, even from normal samples. However, reducing the volumes by 50% resulted in 100% concordance between the visual qualitative diagnoses and the supplier control classifications; visual inspections of results stemming from these modifications were performed by three independent observers and their evaluations were in complete agreement. All field studies were therefore conducted at ambient air incubation temperatures that ranged from 19 to 31 °C using 50% of recommended reaction volumes, with the comparator controls performed at least 3 times daily at different time points during the day.

Briefly, 10 µL of whole blood was added to 200 µL of G6PDH Assay Solution and mixed in a K^+^EDTA microtainer to suspend RBCs. From this reaction volume, 60 µL was spotted onto Whatman 3 MM filter paper following 0, 5 and 10 min of incubation; a further 15 min spot was tested to see whether the absence of stable 37 °C incubation would require longer assay reaction time. After the last time point was spotted, filter papers were air dried for a minimum of 15 min. Fluorescence of NADPH, which is proportional to G6PD activity, was evaluated under long-wave UV light (365 nm). Samples were classified as normal (moderate/strong fluorescence at 5 min, strong fluorescence at 10 min), intermediate (weak fluorescence at 5 min, weak/moderate fluorescence at 10 min), and deficient (faint/no fluorescence at 10 min) by two blinded readers (REH, TF, AR, PAZ), and any discrepancies resolved by discussion with a third adjudicator.

### DNA extraction

Whole blood collected in K^+^EDTA microtainers was stored at 4 °C until DNA extraction could be performed. DNA was extracted from the whole blood samples using the Qiagen QIAmp 96 DNA Blood Kit following recommended protocols with a starting blood volume of 200 µL and an elution volume of 100 µL of Buffer AE (Valencia, CA).

### G6PD PCR amplification

Three primer pairs (IDT, Coralville, IA) were designed to amplify three *G6PD*-specific products. The 812 base pair (bp) exon 2 fragment was amplified using primer set A (Forward A: 5′-AGCATTCCCTGTTTTCCCCA-3′ and Reverse A: 5′-GTGAGACCCCAGAGGAACTC-3′); hg19 nucleotide coordinates 154545441–154546253, GenBank X55448.1 coordinates 3253–4065, GRCh38.p2 coordinates 155316129–155316941. A 3200 bp exon 3–8 fragment was amplified using primer set B (Forward B: 5′-GGTTCTACTGAATTGAATTTTCAGG-3′ and Reverse B: 5′-AGTCTATTCTGATGAACAAGCTGAGG-3′); hg19 coordinates 154533315–154536430, GenBank X55448.1 coordinates 13,075–16,188, GRCh38.p2 coordinates 155304002–155307117. Finally, a 3115 bp exon 9–13 fragment was amplified using primer set C (Forward C: 5′-GTTCATCAGAATAGACTCGAGATGG-3′ and Reverse C: 5′-AACCAGGACAAGAGGACTAAAACC-3′); hg19 coordinates 154530131–154533331, GenBank X55448.1 coordinates 16,172–19,372, GRCh38.p2 coordinates 155300818–155304018. The total length of the human *G6PD* gene analysed in our studies included 7111 bp (812 bp inclusive of exon 2 and flanking intronic sequence [hg19 nucleotide coordinates 154545441–154546253]; 6299 bp inclusive of exons 3–13 and associated intronic sequence [154530131–154536430]).

For exon 2-specific amplification, PCR was performed using the Promega GoTaq^®^ Flexi DNA Polymerase Kit (Madison, WI) in a final reaction mixture of 50 μL; containing 3 μL of genomic DNA, 0.1 μM of each primer, 200 μM of each dNTP, 1.0 mM of MgCl_2_, and 1.25 units of GoTaq DNA polymerase. Amplification conditions were 95 °C for 2 min, followed by 30 cycles of 95 °C for 45 s, 59 °C for 45 s, and 72 °C for 45 s and a final extension at 72 °C for 5 min. For fragments B (exons 3–8) and C (exons 9–13), PCR amplification was performed using the Roche Expand Long Range dNTPack Kit (Indianapolis, IN) in a final reaction mixture of 50 μL; containing 3 μL of genomic DNA, 0.3 μM of each primer (primer set B) or 0.45 μM of each primer (primer set C), 500 μM of each dNTP, 2.5 mM of MgCl_2_, 1.25% DMSO and 3.5 units of Expand Long Range enzyme mix. Amplification conditions were 92 °C for 2 min, followed by 10 cycles of 92 °C for 10 s, 55 °C for 15 s, and 68 °C for 3 min; followed by 25 cycles of 92 °C for 10 s, 55 °C for 15 s, and 68 °C for 3 min increasing 20 s every cycle and a final extension at 68 °C for 7 min.

### G6PD gene sequencing

PCR products were sheared on a Covaris machine for an estimated size of 300 bp. Next, 95 samples were prepared for sequencing according to the Nextera XT DNA Library Preparation Kit (Illumina, Inc., San Diego, CA). Samples were then sequenced on an Illumina MiSeq multiplex run for 2 × 300 bp paired-end reads.

### G6PD sequence data analysis

Illumina MiSeq-generated sequences were first evaluated through FastQC for initial quality control. Sequence reads were trimmed for quality (Q > 20) and adapter sequences using TrimGalore! Those reads that passed the quality filter were aligned to the *G6PD* locus on human reference genome GRCh38 (X:154,526,391–154,552,572) using the Bowtie 2 alignment algorithm [23]. Genotype calls at each base position were determined using mpileup (SAMtools) [24] and custom Perl scripts. Only positions with a coverage greater than 50 reads were considered. Genotypes were then determined based on reference allele frequency, where reference allele frequencies greater than 0.7 were determined to be hemizygous reference, frequencies less than 0.3 were determined to be hemizygous alternate.

### G6PD genotyping

*G6PD* genotyping focused on four single nucleotide polymorphism positions identified through Illumina sequence analysis (202G → A, 376A → G, 563C → T and 1311C → T; nucleotide reference information based on wild-type cDNA sequence; GenBank X03674).

Following PCR amplification, products were further processed by a ligation detection reaction (LDR) that has been described in detail for a variety of additional studies [6, 25–27]. This LDR was performed in a reaction mixture (15 μL) containing 20 mM Tris–HCl buffer (pH 7.6), 25 mM potassium acetate, 10 mM magnesium acetate, 1 mM NAD+ , 10 mM DTT, 0.1% Triton X-100, 13 nM each LDR probe, 1 μL of PCR product, and 2 units of Taq DNA ligase (New England BioLabs, Ipswich, MA). LDR probes consisted of eight allele-specific oligonucleotides and three fluorescently labelled conserved-sequence oligonucleotides. Each allele-specific ‘classification’ probe contained a unique 5′TAG sequence for further hybridization with complementary sequence oligonucleotides bound to Luminex FlexMAP fluorescent microspheres (Luminex Corp., Austin, TX). The conserved-sequence ‘reporter’ probes were phosphorylated at the 5′ end and biotinylated at the 3′ end. Sequences of the classification and reporter probes are provided in Table 1.

**Table 1:** LDR primers and microspheres used to detect G6PD polymorphisms

Polymorphism^a^	LDR primers^b^	Micros^c^
A-	5′-tacaacatctcattaacatatacaAGGCGGGAACGGGCATAGCCCA**C**-3′	37
202G > A	5′-caaacaaacattcaaatatcaatcAGGCGGGAACGGGCATAGCCCA**T**-3′	22
rs1050825	common5′-Phos-GATGAAGGTGTTTTCGGGCAGAA-Biotin-3′	
A	5′-cttaacatttaacttctataacacTGTGACCCCAGGTGGAGGGCAT**T**-3′	30
3769A > G	5′-tacattcaacactcttaaatcaaaTGTGACCCCAGGTGGAGGGCAT**C**-3′	26
rs1050829	common5′-Phos-CATGTGGCTGTTGAGGCGCTGGT-Biotin-3′	
Mediterranean	5′-aatttcttctctttctttcacaatTCTGGTCCTCACGGAACAGGGAG**G**-3′	14
188C > T	5′-cacttaattcattctaaatctatcTCTGGTCCTCACGGAACAGGGAG**A**-3′	28
rs5030868	common5′-Phos-AGATGTGGTTGGACAGCCGGTCA-Biotin-3′	
Silent	5′-cataatcaatttcaactttctactAAGACGTCCAGGATGAGGCGCTC**G**-3′	12
1311C < T	5′-acttatttcttcactactatatcaAAGACGTCCAGGATGAGGCGCTC**A**-3′	34
rs2230037	common5′-Phos-TAGGCGTCAGGGAGCTTCACGTT-Biotin-3′	

^a^Each polymorphic site includes information regarding legacy allele names, cDNA nucleotide change and RefSNP accession ID (rs)

^b^LDR-FMA probes are based on positive-strand sequence. Polymorphic nucleotides are underlined and bold. Lower case letters represent 24 bp tags complementary to unique microsphere anti-tags

^c^Unique microsphere sets designed by Luminex Corp

All post-PCR LDR-FMA methods include the same basic three-step procedure: (i) ligation of specific oligonucleotides to target single- or multiple-nucleotide polymorphisms, (ii) FlexMAP microsphere and streptavidin-R-phycoerythrin (SA:PE) labelling of allele-specific ligation products, and (iii) detection and analysis of the specific fluorescent signals using the BioPlex suspension array system and Bio-Plex Manager analytical software (Bio-Rad Laboratories, Hercules, CA).

Reaction mixtures were initially heated for 2 min at 95 °C, followed by 32 cycles of 95 °C for 15 s and 58 °C for 2 min (annealing and ligation). The LDR product (5 μL) was then added to 60 μL of hybridization solution [3 M tetramethylammonium chloride (TMAC), 50 mMTris–HCl (pH 8.0), 3 mM EDTA (pH 8.0), 0.10% SDS containing Luminex FlexMAP microspheres from each single nucleotide polymorphism (SNP)-specific set (total number of SNP-specific microspheres, n = 8)]. Mixtures were heated to 95 °C for 90 s and incubated at 37 °C for 40 min to allow hybridization between SNP-specific LDR products and microsphere-specific anti-TAG oligonucleotides.

Following hybridization, 6 μL of streptavidin-R-phycoerythrin (Molecular Probes/ThermoFisher) in TMAC hybridization solution (20 ng/μL) was added to the post-LDR mixture and incubated at 37 °C for 40 min in Costar 6511 M polycarbonate 96-well V-bottom plates (Corning). Hybrid complexes consisting of SNP-specific LDR products and microsphere-labelled anti-TAG probes were detected using a Bio-Plex array reader (Bio-Rad Laboratories); the plate temperature was set to 37 °C throughout detection. All fluorescence data were collected using Bio-Rad software, BioPlex Manager 5.0.

## Results

### G6PDd phenotype prevalence

Recruitment of unrelated males in the Ampasimpotsy community (Tsiroanomandidy district) and Mandoto/Miandrivazo districts of Madagascar identified a total of 365 study participants. Mean age (SD) was 23 years (18.7) and 15 years (15.5) in each site, respectively. All were screened for their G6PD phenotype using the FST qualitative test. Overall, 47 study participants (12.9%) were diagnosed as being G6PD deficient. In Mandoto and Miandrivazo districts, 28 of the 230 individuals tested (12.1%) were deficient, with 61% (n = 17) of the deficient having a severe deficiency, and 39% (n = 11) intermediate deficiency. In Ampasimpotsy, 19 of the 135 study participants were G6PD deficient, including 63% (n = 12) severely and 37% (n = 7) intermediate deficient. Overall G6PDd prevalence was not significantly different between the two study populations surveyed (Chi squared test, p = 0.7178).

### G6PD gene sequencing and genotyping

A subset of 95 samples was selected for full *G6PD* gene sequencing following PCR amplification of exons 2–13 and Illumina short-read sequencing. These samples included 15 of the 29 severely deficient, 14 of the 18 intermediate and 66 of the 318 G6PD normal individuals, as diagnosed by the FST. Overall, 16 SNPs were identified across the *G6PD* genomic DNA sequence examined (Table 2). This included six exon-based (three non-synonymous and three synonymous) and ten non-coding SNPs (seven intron and three 3′ non-coding); with the exception of the SNP identified at nucleotide X:154,546,029 (intron 2), all of the SNPs identified in this study had RefSNP accession ID numbers. The six coding region SNPs included nucleotide 202G > A (amino acid 68V > M), 376A > G (126N > D), 563C > T (188S > F), 1116G > A (372Q > Q), 1311C > T (437Y > Y), and 1431C > T (477P > P). The ten non-coding SNPs were identified in introns 2, 5, 7, 8, 11 and in the 3′ untranslated region (Table 2). These SNPs were observed in 20 different haplotypes resulting from Illumina sequencing. The haplotypes included the wild-type *G6PD B* allele and 13 additional variants predicted to share the G6PD normal enzyme activity phenotype. Haplotypes also included phenotypically deficient alleles *G6PD A*-^*202A/376G*^ (two haplotypes), *G6PD A* ^*376G*^ (three haplotypes) and *G6PD Mediterranean* ^*563T*^ (one haplotype) alleles (haplotype details presented in Table 3). Based on these findings an LDR-FMA genotyping assay was developed to evaluate the presence of the identified coding region SNPs for all study samples for which there was sufficient blood available for DNA extraction.

**Table 2:** G6PD single nucleotide polymorphisms (SNPs) identified by Illumina sequencing

X Chromosome: nucleotide coordinate^a^	Ref SNP identifier^b^	Gene position	Coding DNA position^c^	Nucleotide Variation (Ref/Alt)	Minor allele frequency^d^	Codon number and variation
X:154546029		Intron 2		T/G	G = 0.021	
X:154536313	rs762515	Intron 2		T/C	C = 0.221	
X:154536002	rs1050828	Exon 4	202	C/T	T = 0.179	68 V/M^e^
X:154535277	rs1050829	Exon 5	376	T/C	C = 0.211	126 N/D^f^
X:154534699	rs73638302	Intron 5		G/A	A = 0.011	
X:154534556	rs2515904	Intron 5		G/C	C = 0.179	
X:154534419	rs5030868	Exon 6	563	G/A	A = 0.010	188S/F^g^
X:154533860	rs2515905	Intron 7		G/A	A = 0.179	
X:154533413	rs5986990	Intron 8		G/A	A = 0.210	372Q/Q
X:154532738	rs2230036	Exon 10	1116	C/T	A = 0.210	437Y/Y
X:154532439	rs2230037	Exon 11	1311	G/A	A = 0.242	
X:154532293	rs2071429	Intron 11		A/G	G = 0.495	
X:154532214	rs77214077	Exon 12	1431	G/A	A = 0.010	477P/P
X:154531643	rs1050757	3′ UTR		T/C	C = 0.389	
X:154530445	rs12393550	3′ UTR		G/A	A = 0.221	
X:154530400	rs113098908	3′ UTR		G/A	A = 0.210	

^a^Chromosomal coordinates from GRCh38.p2 assembly of the human genome (December 2014)

^b^RefSNP accession ID (rs number) http://www.ncbi.nlm.nih.gov/projects/SNP/snp_ref.cgi?rs=5030872

^c^Originally described cDNA positions are provided to facilitate comparisons across different manuscripts focused on G6PD polymorphisms

^d^Minor allele frequency among the 95 samples evaluated by Illumina sequencing

^e^Also referenced as *G6PD A-* ^*202A*^

^f^Also referenced as *G6PD A* ^*376G*^

^g^Also referenced as *G6PD Mediterranean* ^*563T*^

**Table 3:** G6PD haplotypes and associated phenotypes identified in western Madagascar study sites

Study site	G6PD FST phenotype	Phenotype frequency	*B* ^a^	*A* ^b^	*A-* ^c^	*Mediterranean* ^d^
Ampasimpotsy	Normal	89	88	1	0	0
	Intermediate	7	4	2	1	0
	Deficient	11	1	0	10	0
Mandoto/Miandrivazo	Normal	165	159	6	0	0
	Intermediate	9	6	0	3	0
	Deficient	10	0	0	9	1

Includes samples that were tested by both diagnostic approaches (n = 291)

^a^ *B* wild-type cDNA sequence, GenBank X03674. 84 B haplotypes carry SNP 1311C > T; 437Y > Y

^b^ *A* 126N > D associated with nonsynonymous SNP 376A > G. One A haplotype carries SNP 1311C > T; 437Y > Y

^c^ *A-* 68V > M and 376 N > D associated with nonsynonymous snps 202G > A and 376A > G, respectively. Five A- haplotypes, all from Ampasimpotsy, are 68V > M associated with nonsynonymous snp 202G > A

^d^ *Mediterranean* 188S > F and 437Y > Y associated with nucleotide snps 563C > T and 1311C > T, respectively

Ligation detection reaction-FMA genotyping was applied to 208 samples, including 121 from Mandoto/Miandrivazo and 87 from Ampasimpotsy; 11 samples were analysed by both Illumina sequencing and LDR-FMA (all 11 samples demonstrated 100% concordance between methods). When combined with Illumina sequencing results, 291 individuals were surveyed for *G6PD* DNA sequence polymorphism (Fig. 2). As all individuals studied were males, all results correspond to single haplotypes given male X chromosome hemizygosity. Overall results revealed seven haplotypes characterized by exon-based nucleotide sequence changes across the study sites (Table 3). The majority of the haplotypes were distributed similarly across the study locations, although there was unique distribution of some lower frequency alleles. Of note, the 202A SNP was associated with the 376G among individuals across the Mandoto/Miandrivazo study sites while in Ampasimpotsy, 202A was independent of 376G in 5 of 11 individuals (45.5%). Of further interest, we observed no *G6PD* alleles characteristic of Asian origins or alternative *G6PD A*- mutations observed previously in other African populations (e.g. *G6PD A*-^*680T/376G*^, *G6PD Santamaria* ^*542T/376G*^, *G6PD Betica*-*Selma* ^*968C/376*^ [28]).

### G6PD phenotype-genotype diagnostic concordance

Four of the observed haplotypes (*G6PD B*, *B* ^*1311T*^, *A* ^*376G*^ and *A* ^*376G/1311T*^) were phenotypically normal (95%), while three (*G6PD A*-^*202A/376G*^, *A*-^*202A*^ and *Mediterranean* ^*563T*^) were exclusively associated with a deficient phenotype (Table 3). When *G6PD* haplotypes were compared with FST phenotypes, results were 95.5% concordant. Overall sensitivity of the FST results was 0.951 with one *G6PD B* individual diagnosed as phenotypically deficient and 12 individuals who were genetically normal but characterized with intermediate enzyme activity. The genetically normal but phenotypically deficient individual was diagnosed positive for malaria by RDT at the time of G6PD screening (June 2015). Blinded retesting two months later (August 2015) resulted in a normal G6PD phenotype diagnosis. Specificity of the FST was 1.00 as all 24 individuals with *G6PD*d alleles were diagnosed with intermediate (n = 4) or deficient (n = 20) enzyme activity.

### Malaria prevalence

Across both sites, malaria RDT results were available from 360 participants (Fig. 2). The overall rate of infection was 2.5%, but significantly different across both sites (Chi squared test, p = 0.003), at 0.4% (n = 1/230) in the Mandoto/Miandrivazo population in July 2015 and 6.2% (n = 8/130) from the Ampasimpotsy sample in June and August 2015. There was no significant association between G6PD phenotype status and malaria infection in the sample (Chi squared test, p > 0.5).

## Discussion

The Malagasy population’s origins are unique, though the details relating to the island’s human colonization continue to intrigue and elude archaeologists today [29, 30]. Diverse sources of evidence clearly point to Austronesian origins tracing back to transoceanic trading routes from modern-day Indonesia (specifically, Borneo [31]) and admixture with Bantu communities from continental Africa [32]. For example, the Malagasy language is rooted in the Austronesian languages [33] but also shows the major influence of Bantu dialects [34, 35]. Radiocarbon dating of ancient crop traces suggests that Malagasy crops from the 7th to 12th centuries were of Asian origin [36]; there is also, however, evidence of close ties with the Swahili trading system in western coastal areas [30, 37]. Genome studies are beginning to explore the resulting spatio-genetic structure of the contemporary population [32]. At a coarse level, observations from mitochondrial and Y chromosome studies among Malagasy sub-populations have indicated genetic discontinuity between highland and coastal populations, with highland populations having mainly Indonesian origins and coastal communities being more African [15, 32]. The communities surveyed in the present study were in the highland fringe region (Fig. 1) and self-identified as being predominantly of the Merina highland ethnic group.

The areas selected for this G6PDd screening study were endemic with *P. vivax* transmission. Although the 14 day primaquine regimen is recommended for the radical cure of *P. vivax* by Madagascar’s National Strategic Plan (2013–2017) [38], as in most malaria-endemic regions [12], primaquine is not prescribed. Its numerous limitations as a drug [39] contribute to this, but the potential risk of inducing haemolytic anaemia in patients with a deficiency in glucose-6-phosphate dehydrogenase (G6PDd) enzyme activity levels is the primary limitation. Fears of causing harm by triggering haemolysis, together with logical difficulties of procuring primaquine tables of appropriate posology, have also prevented the application of single-dose treatment to block gametocyte transmission of *P. falciparum*, even in the context of mortal epidemics (Roll Back Malaria committee in Madagascar, November 2016, Pers Comm.). A greater understanding of the relative risks associated with the two applications of primaquine could impact on the burden of malaria in Madagascar. Madagascar is a rare country in 2016 for having substantially increased (>20%) malaria-attributable morbidity and mortality between 2010 and 2015 [1]. All efforts must be made to ensure the toolbox of available interventions is not constrained by poor information. This study, therefore, addresses a key barrier to *P. vivax* radical cure through a study of the epidemiology of G6PDd in a *P. vivax*-endemic zone.

An overall G6PDd phenotype prevalence among males of 12.9% was diagnosed using the fluorescence spot test (12.2% in Mandoto/Miandrivazo and 14.1% in Ampasimpotsy). Global compilations of community-based G6PDd prevalence surveys and of genetic variants have been mapped and published [16, 18]. The methodology followed in the present study was consistent with that of these previous efforts, enabling direct comparisons. The observed G6PDd prevalence in males of 12.9% falls within the 50% confidence interval (11.5–30.3%) of previous predictions based on geostatistical analysis of available population surveys in 2012 [16]. Madagascar was notable in that analysis for being one of the countries with the highest uncertainty surrounding its predicted G6PDd prevalence, emphasizing the need for additional surveys to inform the sparse evidence from the Malagasy population. Setting this result in its geographic context, with the aim of identifying neighbouring areas with potentially similar G6PDd epidemiology, could help to guide programmatic roll-out of single-dose primaquine in Madagascar, a country where no evaluation of this intervention have yet been carried out [12]. The observed G6PDd prevalence in Madagascar was generally consistent with modelled estimates across continental Africa where 15 countries had estimated population-weighted *G6PD*d allele frequencies ≥15%, and prevalence in 19 countries was <10% [16]. Observed prevalence in Madagascar was considerably lower than that estimated from Mozambique, Madagascar’s closest geographic neighbour in continental Africa, which had a modelled predicted prevalence of 21.1% (50% CI 14.7–29.8%) [16]. To the east across the Indian Ocean, Indonesia had a predicted national prevalence of 7.1% (50% CI 5.3–9.4%).

Genetic diversity across the G6PDd Malagasy study population was low, dominated by variants commonly identified among African populations [18]; this was unexpectedly homogenous given the population’s origins. The limited sample size, which was powered to assess phenotypic prevalence, restricted the scope for comprehensive characterization of variant diversity in these populations. However, the lack of an Indonesian footprint on the observed sample was notable. *G6PD*d gene diversity in Indonesian populations is among the greatest globally, including many endemic variants [18]; these did not appear in the Malagasy population screened by the present study. An Indonesian genetic footprint would only be expected to be identifiable if the genetic trait was present in the founding population. Current evidence from genetic studies suggests that the main Indonesian arrival took place in the eighth century CE. This potentially falls after the emergence of some of the most prevalent mutations, estimated to be 1000-6357 years ago for *G6PD A*- [40, 41], 3330 years ago for the *G6PD Mediterranean* ^*563T*^ variant [40], and 1575 years ago for the *G6PD Mahidol* ^*487A*^ variant common across Asia [42]. Though only a small subset of variants, and not including the main Indonesian variants, these dates would suggest that an ancestral Indonesian G6PDd footprint could be expected in the contemporary Malagasy population. A larger number of surveys are required to draw any firm conclusions about an Indonesian contribution to the *G6PD* gene pool in this region of Madagascar.

The present findings carry implications for public health policy. A prevalence of 12.9% G6PDd primarily caused by *G6PD A*-^*202A/376G*^ is similar to that of many sub-Saharan African countries where *P. falciparum* transmission blocking is the main application of primaquine. The WHO has endorsed the use of the single low-dose regimen as a gametocytocide (0.25 mg/kg), a dose considered safe for patients with G6PDd (with the exception of pregnant women, infants aged <6 months and women breastfeeding infants aged <6 months) [43–46]. Certain areas of Madagascar, including the central highlands (from where the Ampasimpotsy population sample originate) and the arid south of the island are prone to local epidemics [2]. In the event of rapidly increasing transmission, the single-dose primaquine regimen could be used in these areas to limit transmission and contribute to curtailing epidemic transmission.

The principal risks with primaquine come from the substantially higher dosing (0.5 mg/kg daily for 14 days) required for the radical cure of *P. vivax*. Given the observed presence of G6PDd in this region, appropriate risk assessments and diagnostic capacity need to be implemented to safely avert morbidity from cumulative relapses of *P. vivax* clinical episodes [47]. While most G6PDd individuals tested for this study carried variants of relatively moderate severity in whom drug-induced haemolysis may be self-limiting [48], there was also a severe mutation observed, *G6PD Mediterranean* ^*563T*^, for whom primaquine-induced haemolysis would be much more dangerous [49] and likely require transfusion to ensure recovery. The WHO guidelines for primaquine and G6PDd screening are deliberately flexible, allowing for differing G6PDd epidemiology and public health infrastructure scenarios. Countries must determine their own risk assessments and reviews of existing experience with primaquine therapy. In the present context of Madagascar, the relatively poorly developed infrastructure for emergency medicine would advise caution regarding the blind use of primaquine. Similarly, given the observation of relatively common (>10%) G6PDd frequency, including occurrence of the severe variant *G6PD Mediterranean* ^*563T*^, it would be strongly advisable to screen all *P. vivax* patients for G6PD enzyme activity prior to administering radical cure doses of primaquine.

The present study has limitations. Anaemia is a known confounder of G6PD screening [17], but was not adjusted for in this study. The high concordance between phenotype and genotype results, however, suggests that this was not a major problem. The limited geographic range of the study, which was restricted to a single malaria transmission ecozone, restricts extrapolation of results across the country; more extensive sampling across different regions would help to gain perspectives on the national-level G6PDd epidemiology as well as the population’s genetic relatedness across the island. The area studied was nevertheless the country’s principal focus of *P. vivax* transmission and hence concerned the population most frequently implicated in potential primaquine drug administration. The present study was limited to male participants. This ensured clearly binary G6PD diagnoses and avoided the spectrum of phenotypes associated with heterozygous *G6PD*d allele carriage. While predictions based on theoretical genetic inheritance principles can estimate frequencies of G6PDd phenotypes and genotypes among females at the population level, large-scale public health programmes increasing access to *P. vivax* radical cure will require the introduction of a quantitative enzyme activity assay that can detect pre-determined acceptable thresholds for safely administering the drug to females [50]. To date, no well-adapted point of care tests suited to this application exist. The 14 days of treatment with primaquine affords opportunities for discontinuing treatment in response to side-effects. In contrast, the drug tafenoquine, which is currently in Phase 3 clinical trials as a single-dose therapy, carries exacerbated risks as a haemolytic agent due to its longer half-life, and will therefore require a G6PDd screening diagnostic with appropriate sensitivity.

## Conclusions

The study presented here explores the epidemiology of G6PDd among previously unstudied populations in *P. vivax*-endemic regions of Madagascar. Prevalence of deficiency being greater than 10% in males carries implications for the administration of primaquine therapy, emphasizing the need for appropriate G6PDd screening ahead of radical cure therapy. The hypothesized *G6PD* genetic diversity anticipated from the diverse origins of the Malagasy population was not reflected in the *G6PD* gene variants despite evidence from other genes, such as the Duffy blood group, suggesting mixed inheritance [6, 8]. More widespread investigations into the epidemiology of G6PDd would provide insight into malaria treatment risk assessments, as well as contribute evidence to ongoing investigations of human archaeology in Madagascar.
